# Disability and ableism: an inconclusive national agenda for the 17th Brazilian National Health Conference

**DOI:** 10.1590/0102-311XEN068723

**Published:** 2023-07-17

**Authors:** Francine de Souza Dias, Martha Cristina Nunes Moreira, Lenir Nascimento da Silva

**Affiliations:** 1 Escola Nacional de Saúde Pública Sergio Arouca, Fundação Oswaldo Cruz, Rio de Janeiro, Brasil.; 2 Instituto Nacional de Saúde da Mulher, da Criança e do Adolescente Fernandes Figueira, Rio de Janeiro, Brasil.

The approach to disability in the field of Public Health has increased in recent decades, in the face of health emergencies and their consequences, as well as the contributions of the social model of disability and the agency of social movements that criticize the exclusively biomedical domain. The relational aspects that constitute the ideals and expectations of capacity come to light, valuing diverse manifestations of human functionality. In this sense, disability is understood as a result of the interaction between body and environment, considering the barriers and human relations that arise in the environment ^1^.

Disability as a human right gains space in the international political agenda by the United Nations, with deep developments in Brazil, which historically follows and normatively incorporates its orientations. This recognition evokes a tension: sometimes people with disabilities are recognized for their potential, sometimes as especially vulnerable ^2^. Theoretical and political efforts ^2^ ^,^ ^3^ agree in the perspective of vulnerability to environmental and relational limitations insufficient to embrace diverse humanity. Approaches that emphasize bodily improvements as a primary strategy to enable collective coexistence, without necessarily disagreeing with environmental and social interventions, are also frequent ^2^ ^,^ ^3^.

Internationally, the political articulation of this group started after the Second World War, with great reach from the 1970s, leveraging the paradigm shift from abnormality to difference. The “first generation” of this mobilization was led by Marxist men with spinal cord injury, concerned with the dimension of access to work for the constitution of independence. The “second generation” of the social model, led by feminists in the 1990s, enhanced the debate by bringing other dimensions of experience to the political scene: gender, pain, care, dependence, race, ethnicity ^3^.

The contribution of black feminism by the paths of intersectionality offers political, theoretical, and methodological tools to face the perception of discrimination as an intersection. In this approach, race, class, gender, and more recently disability and other social markers of difference, are produced and mutually reinforced ^4^ ^,^ ^5^.

This article proposes reflections on the use of these models in the prospection of a Brazilian political agenda re-inaugurated with the new democratic government in 2023, also considering the arrival of the next Brazilian National Health Conference (CNS). Therefore, the preamble was necessary to provoke and enrich the discussions on disability in the Public Health scientific production, contributing to minimize misconceptions in the conceptual approaches. From the notions of “attitudinal barriers” and “ableism”, we enunciate limits, theoretical and ethical-political possibilities to continue an inconclusive debate on health and its inequities revealed in the life of people with disabilities.

Social determinants of health (SDH) and social determination of the health-disease process (SDHDP) - or just social determination - are proposals linked to epidemiology widely disseminated in public health studies. Both are concerned with health inequities, a concept referring to inequalities experienced by different groups, due to socioeconomic, territorial, educational, racial/ethnic, gender, sexual orientation, disability, among other markers, which impact individual and collective health. Despite the nominal similarity, their conceptual divergences have been widely discussed ^6^ ^,^ ^7^ ^,^ ^8^.

The daily difficulties of people with disabilities have been approached mainly in the light of the SDH. This association is based on the conceptual model adopted by the United Nations in 2005. The SDH mark the global health debate and are recognized by the World Health Organization (WHO) ^9^ as “*non-medical factors that influence health outcomes*” in the course of life. International agendas, agreements, and cooperation were activated as strategies to cope with the impacts of this influence.

The main challenges of SDH studies are: establishing a hierarchy that delimits factors of a social, economic, political nature and their agencies, which imply in the health conditions of people and collectives; characterizing SDH individual and group/population, since even if some factors are notable to explain distinctions in singular health states, they may be insufficient to clarify the differences between groups or societies ^10^.

The SDHDP does not focus on hierarchies and takes up sociological aspects to understand how capitalism has produced exploitation, expropriation, violence, subordination, not reducing its effects to mere “factors”. Originally Marxist-based, it understands the discourse of development (mobilizer of the global health agenda), anchored in the inexhaustible production of goods and consumption, as part of the same productive cycle of the deterioration of life and health ^6^. Therefore, the ways of reading the world, facing inequities, and caring in health differ profoundly among the models under discussion.

Unlike racism, a category that names hierarchies based on color, ableism expresses dimensions of hierarchization based on the ruler of body capacity and is a recently constructed category. As Mello ^11^ (p. 3267) explains, ableism is an ideal of functional capacity that produces “*interdiction and biopolitical control of bodies based on the premise of (in)capacity, that is, on what people with disabilities can or are capable of being and doing*”.

Ableism has two inseparable poles: discrimination experienced by people with disabilities and structural logic ^11^ ^,^ ^12^. Both are connected by bodynormativity, which operates molecularly. Like discrimination, it induces subjects considered deviant from the norm to search for a corporal standard functionally recognized as normal ^11^ ^,^ ^12^ and produces subjectivity operating both in the way the people perceive themselves and in the way they are conceived by the other. In this case, the repression of difference with measures of body adjustment and correction are common.

As a structure, ableism evokes “*a bodily and behavioral normativity based on the premise of a total functionality of the individual*” ^12^ (p. 101), producing barriers that can be represented by the most primary social demand of this agenda: accessibility. The production and maintenance of spaces, resources, and relations that make the equal participation of all people unfeasible is the most immediate dimension of ableism as a structure, since it informs the construction of a world based on references that impede, hierarchize, and disqualify dissident bodies. This dimension can be verified in the ideals of productivity, independence, autonomy, performance, and beauty. Otherwise, the judgment of the capacities to exist and of how to exist will necessarily be modulated by an ableist grammar ^13^ and dissociating both dimensions (discrimination and structure) is not possible in the way ableism manifests itself.

The United Nations reiterates that barriers exist as a function of environment and attitudes ^1^. The Brazilian Law of Inclusion (LBI; Art. 3rd, IV, e) ^14^ incorporates attitudes singularly, classifying them as attitudinal barriers: “*attitudes or behaviors that prevent or impair the social participation of people with disabilities in equal conditions and opportunities with other people*”. The same law, when defining discrimination on the grounds of disability, includes practices of restriction, exclusion, action, or omission that harm, impede, or nullify the rights of persons with disabilities, the refusal of reasonable accommodation and assistive technology ^14^. In this case, the inclusion of the attitudinal barrier by the notion of discrimination is perceived. However, in isolation, their weights inform different measures since the idea of attitudinal barrier ignores the modes of operation of bodynormativity.

The global disability agenda is based on the maxim that disability is a development problem, and that poverty and disability are mutually reinforcing ^1^. The association between the two and their mutual constitution are recognized by the approach taken here. However, we conceive disability also as a matter of class, race, ethnicity, gender, generation, which situates the debate in the beginnings and means and not only in the ends. Therefore, evoking theoretical-political tools that help readings and interventions in the meshes of power-knowledge is necessary.

The differences between SDH and SDHDP have been developed for more than a decade and inform different ways of constituting state actions and public policies. Regarding disability, this involves adopting categories that also diverge theoretically, ethically, politically, and technically. Both propositions contribute to the field and can positively affect the living and health conditions of a population, however, there are differences in focus and amplitude.

Thus, we call attention to the way of jointly managing the categories “attitudinal barriers” and “ableism”. We consider that these notions are distinguished in a similar way as the SDH from the SDHDP, which is the starting point of our argumentation. We understand that acting with “attitudinal barriers” and “SDH”, or with “SDHDP” and “ableism” is perfectly possible. However, “SDH” with “ableism” and “SDHDP” with “attitudinal barriers” find theoretical and ethical-political limits. This is due to the values that mobilize each of these formulations revealing different ways of conceiving social relations, human differences, violence, and forms of intervention. Development and social justice are contrasted.

Some of the criticisms related to the SDH are the way this model meets the needs of neoliberal capitalism by not facing the production system and subsidizing the mitigation of its consequences, in addition to denying the contributions of Latin American scholars ^6^. These notes matter due to the way the reading of ableism involves structural dimensions and to the characteristics of Brazilian people with disabilities and the inequities experienced by them, aspects that demand tools more sensitive to the crossing of multiple social markers of difference.

The statement that “*the incidence of the processes of determination implies structural historical modes*” ^6^ (p. 4) corroborates for a better approximation of the SDHDP to discuss inequities in the light of ableism. An interesting approach in this direction is the perspective that health and disability are distinct categories that have as a common dimension the idea of collective, relational production. In addition to being conceived collectively and procedurally, health and disability are crossed by power relations constituted and updated by capitalist production mechanisms, also ableist, racist, and cisheteropatriarchal, hence the need to confront them.

The premise that SDH implies the repetition of the “*modus operandi of causalism: acting on factors and not on the change of structural processes*” ^6^ (p. 5), suggests a certain combination with the perspective of attitudinal barriers, personal improvement services, and improvements in the environment - carried out with governance, economic, social, and management policies. This way of acting aligns with the principles of efficiency, demanding the fragmentation and simplification of reality to carry out direct actions on specific factors, in addition to individual accountability.

Thus, the SDH model contributes to the debate on the social dimensions that cross the health of people with disabilities, without facing the norms and logics that constitute ableism combined with other systems of oppression in the constitution of environments, relationships, policies, and services. Its fecundity with attitudinal barriers is an expression of this disconnect with the production and effects of bodynormativity in the modulation of relations - agencies that classify bodies that escape the expected as sick, making perspectives of other ways of living invisible.

Therefore, advancing in the approaches of the “social” in the scope of public health and in the reflections on the consequences of its use in the production of care is necessary. Social determinants or determination, in isolation, are insufficient models to address and understand the health inequities of people with disabilities, since both have not yet incorporated ableism in their formulations and do not have substantial proximity with the theme, and approaching other areas of knowledge to support reflections related to the theme is necessary. Not making this effort corroborates a pathologized understanding of disability due to the notion of disease.

Thus, these are epidemiological models based on different scientific productions referenced in the Social Sciences, none of them occupied with disability as an analytical category. Therefore, advancing the national agenda entails understanding these distinctions. Such an approach demands dialogue with diverse subjects and with other knowledge, a necessary path to broaden the debate in a critical, qualified, and strengthened way, capable of leveraging the anti-ableist struggle within the scope of the Brazilian Unified National Health System (SUS) and of Public Health, perhaps to make tomorrow a new day, on the eve of the 17th CNS.
